# The views of older women towards mammographic screening: a qualitative and quantitative study

**DOI:** 10.1038/sj.bjc.6605662

**Published:** 2010-05-11

**Authors:** K Collins, M Winslow, M W Reed, S J Walters, T Robinson, J Madan, T Green, H Cocker, L Wyld

**Affiliations:** 1 Centre for Health and Social Care Research, Sheffield Hallam University, Faculty of Health and Wellbeing, 32 Collegiate Crescent, Sheffield S10 2BP, UK; 2 Academic Unit of Supportive Care, Sykes House, University of Sheffield, Little Common Lane, Sheffield S11 9NE, UK; 3 Academic Surgical Oncology Unit, University of Sheffield, K Floor, Royal Hallamshire Hospital, Sheffield S10 2JF, UK; 4 School of Health and Related Research, University of Sheffield, Regent Court, 30 Regent Street, Sheffield S1 4DA, UK; 5 Department of Cardiovascular Sciences (Ageing and Stroke Medicine), University of Leicester, Leicester General Hospital, Gwendolen Road, Leicester LE5 4PW, UK; 6 Academic Unit of Primary Health Care, University of Bristol, Cotham House, Cotham Hill Bristol BS6 6JL, UK; 7 The North Trent Cancer Research Network, Consumer Research Panel, Academic Unit of Supportive Care, Sykes House, Little Common Lane, Sheffield S11 9NE, UK

**Keywords:** breast cancer, oncology, older age, mammographic screening

## Abstract

**Background:**

Mammographic screening has improved breast cancer survival in the screened age group. This improved survival has not been seen in older women (>70 years) where screening uptake is low. This study explores the views, knowledge and attitudes of older women towards screening.

**Methods:**

Women (>70 years) were interviewed about breast screening. Interview findings informed the development of a questionnaire that was sent to 1000 women (>70 years) to quantify their views regarding screening.

**Results:**

Twenty-six women were interviewed and a questionnaire was designed. The questionnaire response rate was 48.3% (479 out of 992). Over half (52.9%, 241 out of 456) of the respondents were unaware that they could request a mammography by voluntary self-referral and were unaware how to arrange this. Most (81.5%, 383 out of 470) had not attended breast screening since turning 70 years. Most (75.6%, 343 out of 454) felt screening was beneficial and would attend if invited. Most (90.1%, 412 out of 457) felt screening should be offered to all women regardless of age or health.

**Conclusions:**

There is a lack of knowledge about screening in older women. The majority felt that invitation to screening should be extended to the older age group regardless of age or health. The current under-utilised system of voluntary self-referral is not supported by older women.

One third of all breast cancers in the United Kingdom are diagnosed in women over 70 years (ONS, 2005). Older women present with the disease at a later stage than their younger counterparts (Diab *et al*, 2000; Wyld *et al*, 2004). The mortality for breast cancer has fallen in most age groups, due both to screening and improved adjuvant therapies. This improved survival has not been seen in older women and mortality is increasing in women over 85 years (DoH, 2009). This may be due to older women being less aware of breast symptoms (Siapush and Singh, 2002), or the lack of screening, both of which may contribute to later diagnosis. In addition, adjuvant chemotherapy and trastuzumab are rarely offered to women over 70 years in the United Kingdom (Cheung *et al*, 2009).

In the United Kingdom, the NHS Breast Screening Programme (NHS BSP) invites all women for triennial mammographic screening between 50 and 70 years of age (extension to age 73 years is planned). Once a woman reaches 70 years, she may continue to have screening, but only if she requests it.

The introduction of breast screening has reduced the death rate from breast cancer by 25–39% in the screened age group, (Tabar *et al*, 2003) with 5-year survival rates for screen detected cancers of 96% compared with 70% for cancer presenting symptomatically (BASO, 2006). However, there is little direct evidence that screening is of benefit to older women. Galit *et al* (2007) reported that screening women aged 75–84 years was associated with a reduced breast cancer mortality. However, others have failed to show this association (Badgwell *et al*, 2008; Schonberg *et al*, 2009). In older women, the influence of competing causes of death may reduce any potential gain from early detection.

However, screening does result in the diagnosis of smaller, earlier stage cancers in older women (Galit *et al*, 2007) and a significantly reduced mastectomy rate (27% screened *vs* 52% symptomatic; Cheung *et al*, 2009).

The evidence is now very compelling that breast screening does overdiagnose cancers: that is it identifies cancers that would not have presented symptomatically in the women's lifetime. The rate of this occurrence is of the order of 25–40% (Morrell *et al*, 2009; Gotzsche *et al*, 2009). For these women, the harms of screening are very real: the unnecessary treatment that may include mastectomy, the use of chemotherapy, radiotherapy and psychological distress (Gotzsche *et al*, 2009).

In the United Kingdom, breast screening uptake in the over 70 years is low with only half of older women aware that they are eligible for screening and only 19% aware of how they can access the service (Kumar *et al*, 2004). Knowledge about breast cancer is also poor in older women (Grunfeld *et al*, 2002), particularly knowledge about breast cancer symptoms and level of risk (Linsell *et al*, 2008). Many older women believe that they are less susceptible than younger women (Mah and Bryant, 1992).

In summary, mammographic screening rates are much lower in women over 70 years, contributing to a later stage at diagnosis and a worse prognosis. At present, we know little about the factors that may prompt older UK women to attend for screening.

The aim of this study was to examine the views, knowledge and attitudes of older women (>70 years) towards mammographic breast screening.

## Materials and methods

Ethics committee approval was obtained. Written informed consent was obtained for each interview. Consent for the questionnaire was presumed if the questionnaire was completed.

The interview schedule and postal questionnaire were developed by the study team and piloted on members of a local Consumer Research Panel (Collins *et al*, 2006) to ensure content and face validity (Ritchie and Spencer, 2003). Both methods explored the participants’ knowledge and views towards breast screening, factors influencing uptake and views regarding the current format and organisation of screening for women over 70 years. The interview themes extracted were used to construct a questionnaire that was extended to a wider population of older women to determine attitudes and correlations with patient characteristics. Most of the questions were constructed as a statement with a Likert format for the response.

### Qualitative interviews

An interview schedule was used to guide discussion to the issues of interest (breast cancer, screening, current and preferred screening arrangements). In-depth, semi-structured interviews were undertaken with 26 purposively selected older women (>70 years) from out patient clinics (surgical, medical, breast) in one UK hospital. Inclusion criteria were the following: female, aged over 70 years, able to read and write in English and able to give informed consent. Exclusion criteria were the following: moderate or severe cognitive impairment.

Recruitment ceased once data saturation had occurred. The interviews were recorded and transcribed. Analysis followed the National Centre for Social Research ‘Framework’ approach to identify recurrent themes (Ritchie and Spencer, 2003). All interviews and data analysis were undertaken by two senior researchers (KC and MW). A thematic index was drawn up and applied to the data. Data were entered into thematic charts and examined to allow interpretation of the data and to identify any relationships between themes.

### Postal questionnaire survey

The questionnaire was devised from themes generated from the qualitative interviews, a systematic literature review and expertise from the study team. The questionnaire comprised 64 questions (with mainly 5-point Likert scale response options) divided into the following sections: demographic details and health characteristics, breast cancer risk factors, past and current breast screening attendance, knowledge and views about breast screening and views about alternative methods of arranging screening for older women. To maximise salience, content and face validity, ease of administration and acceptability of the questionnaire (Boynton and Greenhalgh, 2004), it was piloted on eight members of the North Trent Cancer Research Network Consumer Research Panel (Collins *et al*, 2006). Sampling was through GP practice lists. For the purposes of sample size estimation, we assumed that the primary outcome from the questionnaire survey of older women was to estimate the proportion of women in this age group who would like to have further screening. We assume that this proportion was around 50%, and to estimate this within ±5% (i.e. 95% confidence interval (CI): 45–55%) required around 400 responders to the survey. The questionnaire was distributed to 1000 women, assuming a 40% response rate, to meet the recruitment target of 400 women.

Practices identified all women on the practice list aged over 70 years, but excluded women with significant cognitive impairment. Eligible patients were sent the questionnaire by post. Data were analysed using the Statistical Package for Social Scientists (SPSS version 17) using descriptive data to summarise clinical characteristics of the responders. The *χ*^2^ test was used to examine associations between attitudes to screening and categorical variables (such as age, breast cancer risk category, functional status and number of co-morbidities). Other outcomes from the survey, such as the number and proportion in this age group who would like to have mammograms, were reported along with the associated 95% CI.

## Results

### Qualitative interviews

One hundred and four eligible women were approached for interview and 26 consented (25%). Interview duration ranged from 20 to 60 min. The age range was 70–90 years (median 75 years). All participants were white European. Seven of the 26 women had a history of breast cancer. Fifteen were regular attenders and 11 were non-regular attenders of breast screening up to the age of 70 years. Since reaching 70 years, five women had self-referred for screening. There were no identified variations between the different age subgroups: under 75 years, between 76–85 years or older than 85 years or between women with or without a history of breast cancer.

The thematic frame categorised the data into five themes: breast awareness and behaviour (including risks and benefits), breast screening knowledge and uptake, views about screening over 70 years (important factors influencing women's decisions) and views of the current system of self-referral. The interview findings are presented alongside the questionnaire data below.

### Questionnaire survey

*Respondent characteristics* During 2009, 1000 questionnaires were posted to all eligible women over 70 years registered with four participating GP Practices. The overall response rate to the questionnaire survey was 48.7% (479 out of 983). No reminders were sent out. Therefore, we were unable to compare the characteristics of responders and non-responders in terms of screening views, age or health status. The median age of respondents was 75 years (range 70–95 years). Respondent characteristics are shown in Table 1. No significant differences in responses were found between age cohort and women with or without a history of breast cancer. Most respondents reported having at least one long-term health problem (87.7%, 420 out of 479), 53.2% (255 out of 479) >2 health problems, 27.9% (134 out of 479) 3–4 health problems, 6.5% (31 out of 479) reported ⩾5 health problems. Just over three quarters of the respondents (76.2%, 359 out of 471) were functionally independent.

*Breast awareness and behaviour* The overwhelming message from both the interviews and questionnaires was of a lack of knowledge about both breast cancer and breast screening: in terms of risks, how to access screening and how to examine themselves. Sixty-two percent (297 out of 479; 95% CI: 58–66%) of women indicated that they believed the NHS would have invited them for breast screening over the age of 70 years if it would benefit them.

‘They said to me we ‘shan’t be sending for you again, because we don’t send for people after 65 years old’, and at the time I thought ‘Oh, well fair enough. That must be the time that they think you are out of danger.’ (ID 07, age 75 years)

Most of the women were unaware that breast cancer risk increases with age: 41.5% (193 out of 479) said that they did not know, 34.6% (161 out of 479) thought that the risk was the same and 14.2% (*n*=66 out of 479) thought that the risk was lower in women over 70 years.

‘I have always had the thought, that as you get older these things don’t get hold of you the same…that they’re not likely to kill you the same as a younger person. I don’t know whether that's right or not.’ (ID 03, age 82 years)

Rates of breast self-examination were low, with only 23.6% (112 out of 474) examining themselves regularly compared with 51.7% (*n*=245) occasionally or rarely and 27% (129 out of 478) never. The older women (>85 years) were less likely to examine their breasts (55%) compared with the younger age group 70–74 years (78.4%). Most felt that they did know how to perform self-examination, 80.8% (350 out of 433) having learnt from a range of sources: 58.1% (112 out of 193) by a nurse; 23.3% (45 out of 193) by their GP; 9.3% by a hospital doctor and 9.3% (18 out of 193) through the media, either TV or magazines).

*Breast screening knowledge and uptake* Most respondents (73.1%, 328 out of 449; CI: 69–77%) had regularly attended breast screening when eligible (Table 2), particularly the younger age group (94.4%, 70–74 years). Of the women who had attended routine breast screening 19.6% (74 out of 378) stated that they had been anxious about attending, 44.6% (169 out of 379) stated that the mammogram was uncomfortable, with 23.3% (88 out of 378) describing it as painful. However, despite this, the majority of women (71.2%, 270 out of 446) appreciated the reassurance that they did not have breast disease. Most (81.5%, 383 out of 470; CI: 77.8–84.8%) had not attended breast screening since turning 70 years and the rate fell as age increased (Table 2). Women with a history of breast cancer were significantly more likely to be advised by a HCP to attend breast screening since becoming 70 years (*P*<0.001) than women without a history of breast cancer and more likely to be called back for further tests (*P*<0.001). Reasons for non-attendance are shown in Table 3.

Both interview and survey data suggested that women were uncertain about eligibility for breast screening. Just over half the women (52.9%, 241 out of 456) were unaware that they could request mammography or knew how to access it. Of those that were aware of the service 45.8% (70 out of 153) were told at their previous screening visit, 21.6% (33 out of 153) had heard about it through the media, 13.1% (20 out of 153) had been told by family or friends, 9.2% (14 out of 153) were told by their GP and the remaining 10.6% (16 out of 153) from a combination of the above.

**Figure 1:**
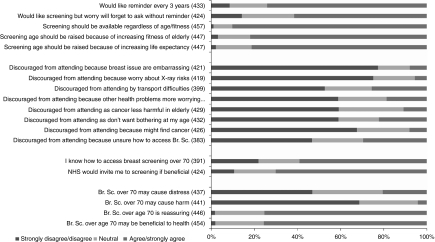
Bar chart showing questionnaire responses relating to attitudes to screening.

*Views about screening in women over 70 years* Most women (75.6%, 343 out of 454; CI: 71.4–79.3%) felt that breast screening was beneficial and would attend if invited. Benefits expressed within the interviews suggested that the most influential factor for attending screening was to increase life expectancy. Women also believed that if breast cancer was detected early, major surgery and longer hospitalisation could be avoided. Women also talked about their wish to maintain optimal quality of life and gain some ‘peace of mind’ knowing that they were clear of breast disease (Figure 1).

‘Just piece of mind really, just the hope that they’re not going to find anything, and if there was anything that they would, that it was early enough, for them to do something about it.’ (ID 15, age 78 years)

In contrast, few women were aware of possible risks of screening (5.5%, 23 out of 419). Despite prompting, most women could only think of the potential radiation risk and transient procedure related pain and discomfort. The survey data found 99.2% (379 out of 382) were not worried by the possible health risks associated with having a mammogram.

Almost two thirds of women (61.6%, 261 out of 424; CI: 56.8–66.0%) said they would forget to attend screening without an invitation with most (74.1%, *n*=321) preferring a reminder letter every 3 years to prompt them to attend.

‘I’m very bad at remembering. If I had a reminder to say go on so and so date I’d be much better at keeping the appointment.’ (ID 01, age 70 years)

Some (25.6%, 102 out of 399) were discouraged from attending because of transport difficulties (either public transport, parking problems) or not wishing to burden family members, 24.7% 104 out of 420).

Within the interviews, several women talked about the generational issue and the embarrassment of being undressed in front of professionals.

‘Women of my age are not accustomed to examining themselves. It's not something that we did… now you see they are telling younger women that they should examine themselves regularly for lumps but we weren’t ever. I doubt whether many of my friends, or anybody of my age does that. It's just not something that you do, it's a bit indelicate...and just this idea of somebody sort of feeling around your breasts...it's a lot to do with your childhood. You mustn’t undress in front of somebody else. You mustn’t let somebody else see your body.’ (ID 30, age 80 years)

However, the survey found that privacy (11.8%, 51 out of 431) or embarrassment (7.8%, 33 out of 421) were relatively uncommon reasons for not wishing to be screened. Significant differences in these attitudinal responses were found between women with or without a history of breast cancer. Women with a history of cancer were less likely to be discouraged from attending screening because of privacy (*P*=0.023) or embarrassment (*P*=0.015).

**Figure 2:**
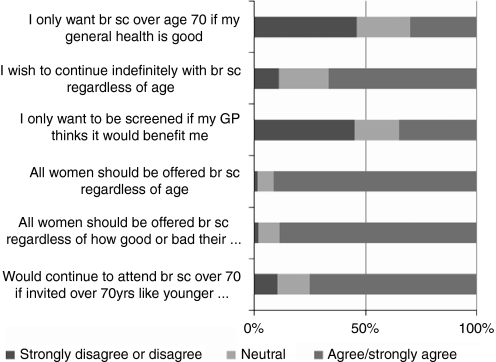
Older women's views about breast screening in women over 70 years.

*Preferences for screening organisation* The overwhelming view across both the interview and questionnaire data was that breast screening should be offered to all women indefinitely and regardless of age, health status or fitness (90.1%, 412 out of 457; CI: 87.0–92.6%). Women did not wish to be exposed to age discrimination (Figure 2). No significant differences in preference responses were found between women with or without a history of breast cancer.

‘It's like being penalised if you’re ill … it's like saying … you’ll probably pop your clogs or something will happen so we’ll not bother calling you.’ (ID 36, age 78 years)

The interviews give greater insight into these areas and emphasised the importance women gave to their right to chose for themselves. They indicated that although they would be willing to discuss the risks and benefits of breast screening with their general practitioner, they would not want them to make decisions on their behalf. They wanted increased information to allow them to make an *informed* decision themselves.

The women interviewed were asked about their views (Figure 2) and preferences (Table 4) of several models of screening service for women over 70 years. Again, this confirms their strong preference for unrestricted screening with 42.9% (178 out of 415), indicating their preference for automatic recall extended indefinitely regardless of age or health status. There was no association between functional status or long-term health problems and the desire to continue to attend breast screening over 70 years if invited. No association was found between individual preferences and functional status, long-term health problems and number of medications taken. Preference for recall options by age are shown in Table 4. There was a trend (*P*=0.044) for women with a history of breast cancer to have a stronger preference for indefinite automatic 3 yearly recall regardless of health status and for GPs to discuss screening and advice, than women without a history of breast cancer.

As indicated in the Introduction, automatic recall for breast screening will extend to age 73 years during 2010 in the United Kingdom. Almost all the women interviewed (25 out of 26) and questionnaire surveyed (90.4%, 377 out of 417) were unaware of this. Although women were generally positive about this extension, overwhelmingly they questioned what the rationale was for only extending the current system by 3 years and why screening was not routinely offered to all women regardless of age.

‘Why 73?…Well you’re on the scrap heap… I think it should be for everybody whatever age, however old, whatever their health.’ (ID 07, age 78 years)

## Discussion

This study has identified important issues in relation to mammographic screening in the over 70-year age group. The use of both qualitative and quantitative methods complemented each other by enabling both an in-depth exploration of the views of this older group of women as well as enabling these issues to be tested and quantified on a larger more generalisible population of older women.

There are several limitations to this study. The study purposively included some women with breast cancer in the interviews to draw out themes of importance to women who had personal experience of the disease. Any bias this may have introduced to the interviews will have been nullified by the larger numeric sampling of the questionnaires, where the incidence of cancer reflected the population norm.

The response rate to the questionnaire was 48%. This questionnaire was distributed through general practitioners to a general population of older women. The study was set up assuming a 40% response rate to meet the recruitment target of 400 women to power statistical analysis and this was achieved. Because of the study design, possible differences between responders and non-responders could not be determined. This introduces the possibility of response bias. It is possible that those who thought screening was a good idea responded, whereas those who were less enthusiastic may not have responded and are therefore not represented in the findings.

GP Practices were selected to be involved in the study on the basis of their patient population being of mixed social, economic and ethnicity. The questionnaire did not specifically ask the women questions relating to their socio-economic status or ethnic groups. However, it is acknowledged that this information would have been helpful in providing useful social and ethnic distribution data.

Although the study aimed to explore the views of women over 70 years, the median age was only 75 years (range 70–95 years). Our data may under-represent the views of the oldest old (over 85 years), where response rates were proportionately lower than expected based on population age distributions. Our sample also had a lower than expected prevalence of women with a history of breast cancer although the proportions of women saying they had regularly attended breast screening between 50 and 70 years of age was similar to the UK National BSP acceptance rates.

Because of the absence of a validated, questionnaire specifically exploring views of mammographic screening in older women, a questionnaire was developed based on the interview data, literature, the expertise within the study team and piloting. The questionnaire did not undergo psychometric testing for reliability and validity. However, face and content validity were ensured by piloting in the target group through qualitative interviewing.

Consistent with earlier research (Linsell *et al*, 2008), breast knowledge and awareness was relatively poor within this study. For example, almost half of the respondents did not know whether the risk of developing breast cancer was higher, lower or the same as younger women. The interviews indicated that only specific breast symptoms such as a lump or tenderness would alert women to seek medical advice. Non-lump symptoms (redness, puckering, change in breast size, nipple discharge) were not viewed as significant. The study also suggests that women remain uncertain about eligibility to attend for breast screening after they become 70 years of age. Very few women could recall receiving information about breast screening either when they attended their last breast screening visit (before reaching 70 years) or in the years following. However, the fact that breast screening was established 20 years ago (extending up to age 65 years initially) means that women over the age of 65 years at the time (and now therefore 85 years) will have had no exposure to screening and therefore might account for the lower levels of knowledge in this age group. At present, when women attend for their final invited screening visit, they should be given information about the availability of screening by self-referral beyond age 70 years. Less than half of the respondents recalled such information being given, suggesting that the present system is ineffective.

Most women had not attended screening since becoming 70 years, and had assumed that breast screening was no longer necessary because they had not received an invitation. However, just over a fifth of women (22.2%) said that they simply ‘did not want bothering’ with breast screening at their age. This is supported by the interview data, which suggested that there was a group of women who did not wish to be screened. The interviews indicated that these women presumed that they were no longer at risk of breast cancer when the recall notices for mammogram ceased.

The majority of women (75.6%) felt that breast screening would be beneficial to their health and would continue breast screening if invited. Almost three in four women surveyed (74.1%) indicated a preference for a postal reminder letter every 3 years. Both interview and survey data suggested that this was because the women were worried they would forget to request an appointment every 3 years. A recent study indicates that reminder letters from the family doctor are effective in increasing screening uptake in the 50–69-year age group (Kaczorowski *et al*, 2009).

The concept of informed choice and a strong desire not to be discriminated against age was evident. However, despite strong views about wanting personal choice, there was also an acknowledgement that some women with debilitating and chronic illness might prefer not to be subjected to further procedures. Current research on how co-morbidity and chronic illness affects screening uptake is variable and conflicting, with some evidence for low screening rates in women with functional impairment (Bynum *et al*, 2005). However, most women in the current study tended not to take into account the influence of co-morbidity and functional limitations because they wished to avoid ageism, similar to earlier findings (Heflin *et al*, 2002). Women seemed unaware of the impact of non-breast major illness on life expectancy and thus, the presence of co-morbid illness did not significantly decrease the desire to be invited for future screening.

As Ramirez (2008) pointed out in their study evaluating psycho-educational interventions to promote early presentation of patients with breast cancer, it is important that women are not made unnecessarily anxious by these interventions. However, with regard to promoting levels of awareness of the self-referral system in women over 70 years, future work evaluating the effectiveness of targeted information is urgently needed as the present system is clearly ineffective.

The International Society for Geriatric Oncology recommends screening be available up to age 75 years, with individualised decision making beyond this based on patient preference, physiological age and life expectancy (Wildiers *et al*, 2007). The planned extension of the NHS BSP upper age limit to 73 years, will put the United Kingdom more in line with this, but steps need to be taken to educate older women about the availability of screening on demand beyond this age, so that they can make an informed choice about whether to continue to attend.

In conclusion, the study indicates a lack of knowledge about breast cancer diagnosis and uncertainty and confusion about eligibility to attend for screening. There may be a need to consider providing up-to-date high-quality targeted information regarding breast screening for women over age 70 years, to enable informed choice about attending for mammographic screening. The study also shows the reluctance of patients to have their general practitioner acting as a gatekeeper to access to mammography screening. The prevailing view expressed being that that this group of women wanted increased information about the benefits and risks of screening to feel able to make their own *informed* decision as to whether they would wish to attend. It denoted the importance these women placed on their perceptions of self-worth, of feeling that they are still significant in society whatever be their age. The study indicates the need for improved delivery and dissemination of information to improve knowledge and awareness of the risks and benefits and the availability of screening. The current system of voluntary self-referral does not seem to be appropriate for this age group.

## Figures and Tables

**Table 1:** Characteristics of respondents

	**Sample**		**Population**	
**Characteristic**	***n***	**%**	**%**	**Data source**
*Patient age group (years)*				
70–74	200	43	30	ONS, 2007^a^
75–79	134	29	27	
80–84	71	15	21	
85+	60	13	22	
Total	465	100	100	
				
*Lives alone*				
No	275	58		
Yes	202	42		
Total	477	100		
				
*Functional status*				
Independent	359	76		
Dependent	112	24		
Total	471	100		
				
*Any long-term health problem*				
No	59	12		
Yes	420	88		
Total	479	100		
				
*Any tablets or medicines taken regularly*				
No	53	11		
Yes	426	89		
Total	479	100		
				
*Previously had breast cancer*				
No	434	95		
Yes	23	5	12	Can Res UK, 2008^b^
Total	457	100		
				
*Any relatives had breast cancer*				
No	333	77		
Yes	97	23		
Total	430	100		
				
*Do you examine your breasts*				
No	129	27		
Yes	349	73		
Total	478	100		
				
*Attended breast screening regularly between 50 and 70 years of age*				
No	121	27		
Yes	328	73	73	NHS BSP, 2007/8^c^
Total	449	100		

a ONS Mid-2007 Population Estimates: England; estimated resident population by single year of age and sex.

b Cancer Research UK. Approximately 12% of the UK female population aged ⩾65 years have had a diagnosis of breast cancer. Source: http://info.cancerresearchuk.org/cancerstats/types/breast/incidence/#prev.

c NHS Breast Screening Programme Annual Review 2009. Breast screening acceptance rates in 2007/8 for women aged 50–70 years.

**Table 2:** Past and current breast screening attendance by age cohort

	**Patient age group (years)**								**Total**	
	**70–74**		**75–79**		**80–84**		**85+**			
	***n***	**%**	***n***	**%**	***n***	**%**	***n***	**%**	***n***	**%**
*Attended breast screening regularly between 50 and 70 years of age*										
No	11	5.6	15	12.4	42	66.7	49	87.5	117	26.8
Yes	186	94.4	106	87.6	21	33.3	7	12.5	320	73.2
Total	197	100.0	121	100.0	63	100.0	56	100.0	437	100.0
										
*Never attended breast screening*										
Attended	198	99.0	132	98.5	46	64.8	21	35.0	397	85.4
Never	2	1.0	2	1.5	25	35.2	39	65.0	68	14.6
Total	200	100.0	134	100.0	71	100.0	60	100.0	465	100.0
										
*Attended breast screening since aged 70 years because I was asked to attend myself*										
No	153	79.3	96	77.4	61	92.4	53	98.1	363	83.1
Yes	40	20.7	28	22.6	5	7.6	1	1.9	74	16.9
Total	193	100.0	124	100.0	66	100.0	54	100.0	437	100.0
										
*Health care professional advised me to attend breasting screening since becoming 70 years*										
No	176	95.1	113	94.2	64	97.0	52	94.5	405	95.1
Yes	9	4.9	7	5.8	2	3.0	3	5.5	21	4.9
Total	185	100.0	120	100.0	66	100.0	55	100.0	426	100.0
										
*If attended breast screening were you called back for further tests?*										
No	164	83.2	113	87.6	63	92.6	51	89.5	391	86.7
Yes	33	16.8	16	12.4	5	7.4	6	10.5	60	13.3
Total	197	100.0	129	100.0	68	100.0	57	100.0	451	100.0

**Table 3:** Reasons for screening non-attendance in women over 70 years

**Reasons given for non-attendance to breast screening >70 years**	***n* (%)**
Not invited for screening so thought not necessary (*n*=382)	199 (52.1)
Did not know I could refer myself (*n*=382)	134 (35.1)
Felt mammograms not needed at my age (*n*=382)	72 (18.8)
Other health problems seem more important (*n*=383)	66 (17.2)
I did not want any more mammograms (*n*=382)	47 (12.3)
I forgot about it (*n*=382)	35 (9.2)
Mammograms painful/unpleasant (*n*=382)	17 (4.5)
Worried about getting to screening centre (*n*=382)	15 (3.9)
Worried about the risks of having mammograms (*n*=382)	3 (0.8)

**Table 4:** Preference for different models proposed

	**Patient age group (years)**									
	**70–74**		**75–79**		**80–84**		**85+**		**Total**	
**Preference for breast screening service**	***n***	**%**	***n***	**%**	***n***	**%**	***n***	**%**	***n***	**%**
Maintenance of the current system, where women over 70 years are not called back routinely but can request screening themselves	10	6.1	24	25.3	12	24.5	6	17.6	52	15.2
Automatic 3 yearly call-back letters inviting women for screening to be extended up to age 75 years, regardless of general health	34	20.6	8	8.4	4	8.2	1	2.9	47	13.7
Selected call-back for fitter women only, after age 70 years, depending on whether they have other illnesses and therefore might not benefit from screening	3	1.8	4	4.2	2	4.1	2	5.9	11	3.2
Automatic 3 yearly call-back letters, inviting women for screening, to be extended indefinitely, regardless of health status	99	60.0	50	52.6	16	32.7	10	29.4	175	51.0
GPs to discuss screening with older women at their health check and advise whether to continue to attend for breast screening	19	11.5	9	9.5	15	30.6	15	44.1	58	16.9
										
Total	165	100.0	95	100.0	49	100.0	34	100.0	343	100.0

## References

[bib1] Badgwell BD, Giordano SH, Duan ZZ, Fang S, Bedrosian I, Kuerer HM, Singletary E, Hunt KK, Hortobagyi GN, Babiera G (2008) Mammography before diagnosis among women age 80 years and older with breast cancer. Eur J Cancer Clin Oncol 26: 2482–2488 10.1200/JCO.2007.12.8058 18427152

[bib2] BASO (2006) NHS Breast Screening Programme and Association of Breast Surgery at the British Association of Surgical Oncology. An audit of screen detected breast cancers for the year of screening April 2005 to March 2006. http://www.cancerscreening.nhs.uk/breastscreen/publications/baso2005--2006.pdf

[bib3] Boynton PM, Greenhalgh T (2004) Selecting, designing, and developing your questionnaire. Br Med J 328: 1312–1315 15166072 10.1136/bmj.328.7451.1312 PMC420179

[bib4] Bynum JPW, Braunstein JB, Sharkey P, Kathleen H, Wu AW (2005) The influence of health status, age, and race on screening mammography in elderly women. Arch Intern Med 165: 2083–2088 16216997 10.1001/archinte.165.18.2083

[bib5] Cheung S, Greenway N, Lagord C, Williams L, Kearins O, Lawrence G (2009) All Breast Cancer Report. A UK analysis of all symptomatic and screen-detected breast cancers diagnosed in 2006. West Midlands Cancer Intelligence Unit: Birmingham, UK, pp 1–45

[bib6] Collins K, Stevens T, Ahmedzai SA (2006) Can Consumer Research Panels form an effective part of the Cancer research Community. J Clin Effectiveness Nurs 9: 112–118

[bib7] Department of Health (DoH) (2009). Cancer Reform Strategy: achieving local implementation-second annual report. Produced by COI for The Department of Health Cancer Programme: London

[bib8] Diab SG, Elledge RM, Clarke GM (2000) Tumour characteristics and clinical outcome of elderly women with breast cancer. J Natl Cancer Inst 92: 550–556 10749910 10.1093/jnci/92.7.550

[bib9] Galit W, Green MS, Lital KB (2007) Routine screening mammography in women older than 74 years: a review of the available data. Maturitas 57: 109–119 17336004 10.1016/j.maturitas.2007.01.010

[bib10] Gotzsche PC, Hartling O, Nielson M, Brodersen J, Jorgensen KJ (2009) Breast screening: the facts or maybe not. Br Med J 338: 86 10.1136/bmj.b86 19174442

[bib11] Grunfeld EA, Raminez AJ, Hunter MS, Richards MA (2002) Women's knowledge and beliefs regarding *breast cancer*. Br J Cancer 86: 1373–1378 11986766 10.1038/sj.bjc.6600260 PMC2375381

[bib12] Heflin MT, Oddone EZ, Pieper CF, Burchett BM, Cohen HJ (2002) The effect of comorbid illness on receipt of cancer screening by older people. J Am Geriatr S 50: 1651–1658 10.1046/j.1532-5415.2002.50456.x 12366618

[bib13] Kaczorowski J, Karwalajtys T, Lohfeld L, Laryea S, Anderson K, Roder S, Sebaldt RJ (2009) Women's views on reminder letters for screening mammography. Can Fam Physician 55: 622–623 19509209 PMC2694090

[bib14] Kumar ID, Reed MWR, Wyld L (2004) Breast screening in the older woman. Efficacy and awareness of availability. Eur J Surg Oncol 30: 1012

[bib15] Linsell L, Burgess CC, Ramirez AJ (2008) Breast Cancer awareness among older women. Br J Cancer 99: 1221–1225 18813307 10.1038/sj.bjc.6604668 PMC2570528

[bib16] Mah Z, Bryant H (1992) Age as a factor in breast cancer knowledge, attitudes and screening behaviour. Can Med Assoc J 146: 2167–2174 1308756 PMC1492292

[bib17] Morrell S, Barratt A, Irwig L, Howard K, Biesheuvel C, Armstrong B (2009) Estimates of over diagnosis of invasive breast cancer associated with screening mammography. Cancer Cases Control 21(2): 275–282 10.1007/s10552-009-9459-z 19894130

[bib18] Office for National Statistics (2005) Cancer Registration Statistics for England 2005. Office for National Statistics: London

[bib19] Ramirez AJ (2009) Promoting early presentation of breast cancer by older women: a preliminary evaluation of one-to-one health professional-delivered intervention. J Psychosom Res 67: 377–387 19837200 10.1016/j.jpsychores.2009.01.005

[bib20] Ritchie J, Spencer L (2003) Carrying out qualitative analysis. In Qualitative Research Practice. Ritchie J, Lewis J (eds) pp 219–262. Sage Publications: London

[bib21] Schonberg MA, Silliman RA, Marcantonio ER (2009) Weighing the benefits and burdens of mammography screening among women age 80 years or older. Am J Clin Oncol 27: 1774–1780 10.1200/JCO.2008.19.9877 PMC2668704 19255318

[bib22] Siapush M, Singh G (2002) Sociodemographic variations in breast cancer screening behaviour among Australian women: results from the 1995 national health survey. Prev Med 35: 174–180 12200103 10.1006/pmed.2002.1063

[bib23] Tabar L, Yen MF, Vitak B, Chen THH, Smith RA, Duffy SW (2003) Mammography service screening and mortality in breast cancer patients: 20-year follow-up before and after introduction of screening. Lancet 361: 1405–1410 12727392 10.1016/S0140-6736(03)13143-1

[bib24] Wildiers H, Kunkler I, Biganzoli L, Fracheboud J, Vlastos G, Bernard-Marty C, Hurria A, Extermann M, Girre V, Brain E (2007) Management of breast cancer in elderly individuals: recommendations of the International Society of Geriatric Oncology. Lancet Oncol 8: 1101–1115 18054880 10.1016/S1470-2045(07)70378-9

[bib25] Wyld L, Garg DK, Brown H, Reed MWR (2004) Stage and treatment variation with age in postmenopausal women with breast cancer: compliance with guidelines. Br J Cancer 90: 1486–1491 15083173 10.1038/sj.bjc.6601742 PMC2409727

